# Impact of rifaximin use in infections and mortality in patients with decompensated cirrhosis and hepatic encephalopathy

**DOI:** 10.1177/17562848241254267

**Published:** 2024-05-27

**Authors:** Francisco Idalsoaga, Camila Robles, Andrea Ortiz, Oscar Corsi, Eduardo Fuentes-López, Luis Antonio Díaz, Gustavo Ayares, Marco Arrese, Juan Pablo Arab

**Affiliations:** Departamento de Gastroenterología, Escuela de Medicina, Pontificia Universidad Católica de Chile, Santiago, Chile; Departamento de Medicina Interna, Escuela de Medicina, Pontificia Universidad Católica de Chile, Santiago, Chile; Departamento de Medicina Interna, Escuela de Medicina, Pontificia Universidad Católica de Chile, Santiago, Chile; Departamento de Gastroenterología, Escuela de Medicina, Pontificia Universidad Católica de Chile, Santiago, Chile; Departamento de Ciencias de la Salud, Facultad de Medicina, Pontificia Universidad Católica de Chile, Santiago, Chile; Departamento de Gastroenterología, Escuela de Medicina, Pontificia Universidad Católica de Chile, Santiago, Chile; Departamento de Gastroenterología, Escuela de Medicina, Pontificia Universidad Católica de Chile, Santiago, Chile; Departamento de Gastroenterología, Escuela de Medicina, Pontificia Universidad Católica de Chile, Santiago, Chile; Division of Gastroenterology and Hepatology, Department of Medicine, Schulich School of Medicine, Western University and London Health Sciences Centre, University Hospital, 339 Windermere Road, Room A10-224, London, ON N6A 5A5, Canada; Department of Epidemiology and Biostatistics, Schulich School of Medicine, Western University, London, ON, Canada

**Keywords:** cirrhosis, hepatic encephalopathy, infections, rifaximin

## Abstract

**Introduction:**

Infections in patients with cirrhosis are associated with high morbidity and mortality. Rifaximin is an antibiotic used to treat and prevent hepatic encephalopathy (HE); however, it has been suggested that it may play a crucial role in reducing infections in these populations.

**Aim:**

To evaluate the role of rifaximin in preventing frequent cirrhosis-related infections [spontaneous bacterial peritonitis, pneumonia, urinary tract infection (UTI), and bacteremia], *Clostridioides difficil*e infection, and all-cause mortality, as well as determining adverse effects and adherence to the drug.

**Methods:**

A retrospective cohort study was conducted on decompensated cirrhotic patients with history of HE between January 2017 and November 2022 at a university center. Patients with cirrhosis, regardless of their etiology and severity, were included in the study, encompassing both hospitalized and outpatient cases. The statistical analysis included adjusted general linear models, Poisson regressions, and propensity score matching.

**Results:**

We included 153 patients. The mean age in the cohort was 60.2 ± 12.3 years and 67 (43.8%) were women. The main cause of cirrhosis was metabolic dysfunction-associated steatotic liver disease 52 (38%), and the median Model of End-Stage Liver Disease sodium was 16.5 (7–32). In the cohort, 65 (45%) patients used rifaximin. The mean follow-up was 32 months. Eighty-five patients with infectious events were recorded, and a total of 164 infectious events were registered. The main infectious events were UTIs (62, 37.8%) and pneumonia (38, 23.2%). The use of rifaximin was associated with lower infection rates, displaying an incidence rate ratio (IRR) of 0.64 [95% confidence interval (CI) (0.47–0.89); *p* = 0.008]. However, no discernible impact on mortality outcome was observed [IRR 1.9, 95% CI (0.9–4.0); *p* = 0.09]. There were no reported adverse effects, and no patient discontinued the therapy due to adverse effects.

**Conclusion:**

The use of rifaximin significantly reduces infections in patients with cirrhosis and HE. Despite rifaximin was associated with a decreased all-cause mortality, this impact was not statistically significant in the adjusted analysis.

## Plain language summary

Assessing the impact of rifaximin on infections in cirrhosis

This study aimed to investigate the role of rifaximin, an antibiotic commonly used to treat hepatic encephalopathy, in preventing infections and mortality in patients with cirrhosis. The retrospective cohort study included 153 decompensated cirrhotic patients with a history of hepatic encephalopathy, covering the period from January 2017 to November 2022 at a university center. Results showed that 45% of the patients in the cohort used rifaximin, and the mean follow-up duration was 32 months. A total of 164 infectious events were recorded during the study, with urinary tract infections (37.8%) and pneumonia (23.2%) being the most common. The use of rifaximin was associated with a significant reduction in infection rates, with an incidence rate ratio of 0.64 (95% CI [0.47-0.89]; p=0.008). However, there was no statistically significant impact on all-cause mortality (IRR 1.9, 95% CI [0.9-4.0]; p=0.09). Notably, no adverse effects were reported, and no patient discontinued rifaximin therapy due to adverse effects. In conclusion, rifaximin demonstrated a noteworthy reduction in infections among cirrhotic patients with hepatic encephalopathy. Although a decrease in all-cause mortality was observed with rifaximin use, this effect did not reach statistical significance in the adjusted analysis. The study supports the potential benefits of rifaximin in preventing infections in this vulnerable patient population without apparent adverse effects. Further research may provide additional insights into the long-term impact of rifaximin on mortality outcomes in cirrhotic patients.

## Graphical abstract


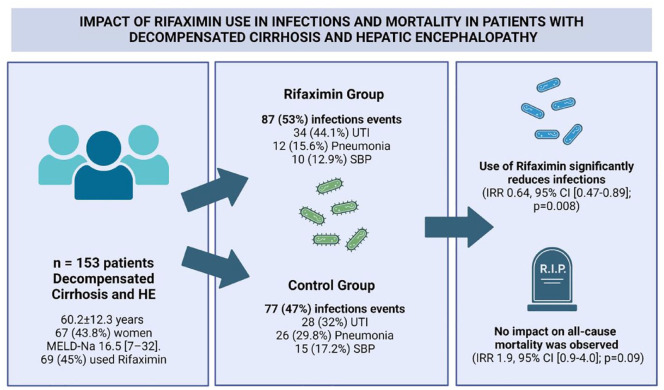


## Introduction

Cirrhosis is a growing cause of morbidity and mortality in the most developed countries, being the 14th most common cause of death worldwide and the fourth one in Central Europe.^1^ It is associated with multiple life-threatening complications, including hepatic encephalopathy (HE), ascites, esophageal variceal bleeding (EVB), hepatorenal syndrome (HRS), spontaneous bacterial peritonitis (SBP), among others.^2^ In particular, bacterial infections are common in cirrhotic patients, reaching a prevalence of about 25–47% among hospitalized cirrhotic patients.^3,4^ Infections also constitute a significant cause of hospitalization in cirrhotic patients, accounting for admission in 30–50% of cases.^5^ The high prevalence of infections results in increased healthcare costs and a greater burden on healthcare services.^3^ Infections not only impose a significant burden on healthcare systems but also have a pivotal impact on the prognosis of cirrhotic patients, increasing their morbidity and mortality.^1,6^ Among the infections, urinary tract infections (UTIs), pneumonia, bacteremia, and SBP have been reported as the main causes of infection in these patients.^7^

Preventing infections has an individual impact on patients, as well as the prevention of antibiotic resistance. Unfortunately, antibiotic overuse is associated with adverse effects and a rising prevalence of antibiotic resistance. This is particularly important in cirrhotic patients due to their high rate of hospitalizations, increased susceptibility to infections,^8^ and frequent use of antibiotic treatments. High rates of multiple antimicrobial resistance, including quinolones (up to 40%), have been reported in patients with SBP who receive prophylactic antibiotics.^9^ Furthermore, global studies describe a prevalence of multidrug-resistant bacteria in cirrhotic patients of 23–34%, with significant variations by geographical region, being more prevalent in Asia.^4,10,11^

Rifaximin is a broad-spectrum antibiotic with minimal gastrointestinal absorption,^12,13^ commonly used as part of therapy for HE. In this context, the use of rifaximin has proven effective in managing HE, leading to a reduction in hospitalizations, shorter hospital stays, and even a decrease in mortality when compared to the use of lactulose alone in patients with HE.^13–15^ It has also proven effective in the prevention and treatment of this decompensation.^12^ Immunomodulatory effects of rifaximin within the intestinal microbiota have been reported, potentially reducing the risk of SBP, *Clostridioides difficile* diarrhea, and possibly other infections.^14,16,17^ Furthermore, this antibiotic has been investigated for its role in preventing other complications of cirrhosis, including EVB, refractory ascites, hepatorenal syndrome (HRS), hospital readmission, and transplant-free survival.^12,16,18,19^ The use of rifaximin is limited in some regions, as is the case in Latin America, due to the associated costs of this medication and the varying healthcare system coverage.^20^ There is limited data in Latin America regarding rifaximin and its associated benefits in decreasing infections. Therefore, this study aims to evaluate the role of rifaximin in preventing common cirrhosis-related infections (SBP, pneumonia, UTIs, and bacteremia) and *C. difficile* infection, assessing the impact of this therapy on mortality in Hispanic patients.

## Methods

### Study design and participants

We conducted a retrospective registry-based study of patients with decompensated cirrhosis and history of HE. We included all cirrhotic patients with a history of HE who were either undergoing follow-up at the Hepatology/Pre-Liver Transplant clinic or were hospitalized at the UC-CHRISTUS Clinical Hospital in Santiago, Chile, between January 2017 and November 2022. Patients with cirrhosis were included regardless of their etiology and severity, as well as irrespective of the admission unit (general ward, intermediate care unit, or critical care unit). The diagnosis of cirrhosis was made based on the clinical history and imaging by the attending physician. A liver biopsy was not mandatory for inclusion and was considered only in clinical uncertainty by the attending physician. We excluded patients under 18-year-old, pregnant women, patients with use of immunosuppressant drugs (different from those indicated for liver diseases), hepatocellular carcinoma (end-of-follow-up or admission exclusion criteria), advanced or metastatic local stage neoplasia, and hematologic cancers. To evaluate the impact of using rifaximin, only those patients who had used rifaximin for at least 2 weeks prior to the infectious event were considered.

### Data collection

We used a de-identified electronic spreadsheet database to collect data from patients with the aforementioned criteria. We obtained demographic, clinical, and analytical data recorded at the time of study entry. We also collected data on rifaximin usage, which included dosage information, reports of adverse effects, and data pertaining to patient adherence. Mortality was assessed using the publicly available Chilean national registry. The history of alcohol consumption was extracted from clinical records. The criteria set by the National Institute on Alcohol Abuse and Alcoholism were employed to define moderate and heavy alcohol consumption. In women, moderate intake is defined as consuming less than three drinks on any single day and no more than seven drinks per week. For men, it is defined as consuming no more than 4 drinks on any single day and no more than 14 drinks per week. Heavy intake is considered for women when they consume 4 or more drinks on any day or 8 or more drinks per week, and for men, it is when they consume 5 or more drinks on any day or 15 or more drinks per week.^21^

### Outcome

The main outcome of the study was to evaluate the effect of the use of rifaximin on the incidence of infections and mortality. The development of SBP, pneumonia, bacteremia, UTI, and diarrhea due to *C. difficile* was exclusively evaluated as the infections of interest. The safety objective is to identify the frequency of adverse effects with the use of rifaximin.

### Statistic analysis

Continuous data were described using mean and standard deviation (SD) or median and interquartile ranges for those variables without normal distribution. Normal distribution was evaluated using the Shapiro–Wilk test. Nominal data were described using percentages. To compare numerical variables with normal distribution, Chi-squared test, and Student’s *t* test or analysis of variance were used. For numerical variables that do not have a normal distribution, non-parametric tests were used. In the case of multiple proportions, the *p* value was estimated using binomial regression with a log link function.

For the analysis of the incidence of infections, the Poisson regression was used at the beginning because it is a discrete count of events, and the result was expressed in the risk index ‘incidence rate ratio’ (IRR), with the corresponding 95% confidence interval. An initial non-adjusted analysis and another one adjusted for the confounding variables found related to infections and use of rifaximin were performed, within which gastrointestinal bleeding, alcohol consumption, initial MELD, and initial Child–Pugh were found. Subsequently, propensity score matching was used. Firstly, evaluating the probability of being treated and the goodness-of-fit of the predictions (if goodness-of-fit is adequate, the model predictions for the probability of being treated are satisfactory). Then, propensity score quintiles were generated for a careful adjustment of the confounding variables, and the balance between both groups was checked. Prior to the use of propensity, the groups were not homogeneous, so its use was justified. After propensity score adjustment, the groups were balanced. Finally, the stratification by quintiles of the propensity score was included in the Poisson model and adjusted models were obtained by confounders described in the literature for the variables of HE, infections, and use of rifaximin. To check for consistency of the effect within the Child–Pugh groups, a heterogeneity analysis was conducted using the ‘Rifaximin-Child–Pugh interaction’ variable in a multivariate analysis. The results showed a non-statistically significant interaction [95% confidence interval (CI) (0.7–1.7); *p* = 0.58], indicating no significant heterogeneity among the groups. We assessed transplant-free survival and infection-free survival through Kaplan–Meier analysis. Subsequently, mortality was analyzed using a non-adjusted generalized linear model (GLM) with adjustments for baseline and Child–Pugh. The statistical analysis was carried out using STATA software version 17.0 (StataCorp LP, College Station, TX, USA). A *p* value of <0.05 was considered statistically significant.

### Ethical considerations

The study protocol was approved by the Scientific Ethics Committee of Health Sciences of the Pontificia Universidad Católica de Chile, and a waiver of informed consent was obtained for the review of clinical records (ID: 210623028).

## Results

### Baseline characteristics of the cohort

**Figure 1.**
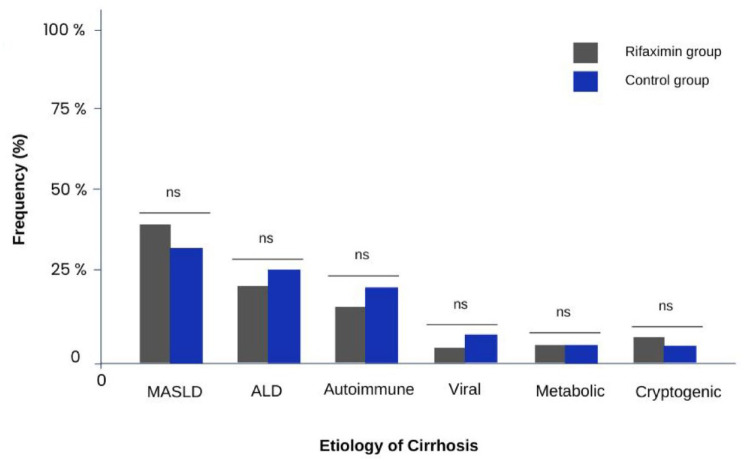
Etiology of cirrhosis. The graph shows the percentage of patients according to the etiology of their cirrhosis at admission. ALD, alcohol-associated liver disease; MASLD, metabolic dysfunction-associated steatotic liver disease.

We included 153 patients. The mean age in the cohort was 60.2 ± 12.3 years and 67 (43.8%) were women (Table 1). The main causes of cirrhosis were metabolic dysfunction-associated steatotic liver disease (MASLD) in 52 (38%) cases, alcohol-related liver disease in 36 (23.5%), autoimmune hepatitis in 23 (15%), and chronic viral hepatitis infection (including Hepatitis B and Hepatitis C) in 6 (3.9%) (Figure 1). In the overall cohort, the median Model of End-Stage Liver Disease sodium (MELD-Na) was 16.5 (7–32), while 42 (27.5)% were Child–Pugh A, 60 (39.2%) were Child–Pugh B, and 51 (33.3%) were Child–Pugh C. Of note, 138 (90.2%) of patients had history of ascites, 85 (55.6%) overt HE, 115 (75.2%) esophageal varices, 45 (29.4%) EVB, 15 (9.8%) portal vein thrombosis, and 15 (9.8%) hepatocellular carcinoma.

**Table 1.** Baseline characteristics of patients according to the use of rifaximin.

Characteristics	Global (*N* = 153)	Rifaximin group (*N* = 69)	Control group (*N* = 84)	*p* Value
Age (mean)	60.2 ± 12.3	60.1 ± 9.3	60.2 ± 14.3	0.93
Sex				
Female	67 (43.8%)	31 (44.9%)	36 (42.9%)	0.79
Male	86 (56.2%)	38 (55.1%)	48 (57.1%)	
Etiology				
MASLD	52 (33.9%)	28 (40.6%)	24 (28.6%)	0.28
ALD	36 (23.5%)	15 (21.7%)	21 (25.0%)	0.71
Autoimmune	23 (15.0%)	7 (10.1%)	16 (19.0%)	0.18
Viral (Hepatitis B and C)	6 (3.9%)	1 (1.4%)	5 (6.0%)	0.16
HH and Wilson disease	7 (4.6%)	3 (4.3%)	4 (4.8%)	0.60
Cryptogenic	6 (3.9%)	4 (5.8%)	2 (2.4%)	0.78
Others	23 (15.0%)	11 (15.9%)	12 (14.3%)	0.68
Child–Pugh (*N*, %)				
A	42 (27.5%)	18 (26.1%)	24 (28.6%)	0.79
B	60 (39.2%)	25 (36,2%)	35 (41.7%)	0.65
C	51 (33.3%)	26 (37.7)	25 (29.8%)	0.46
MELD-Na	16.5 (7–32)	16.7 (8–30)	16.4 (7–32)	0.72
History of decompensation (*N*, %)				
Ascites	138 (90.2%)	66 (95.7%)	72 (85.7%)	0.04
Spontaneous bacterial peritonitis	17 (11.1%)	11 (15.9%)	6 (7.1%)	0.08
Overt hepatic encephalopathy	85 (55.6%)	51 (73.9%)	34 (40.5%)	<0.001
Esophageal varices	115 (75.2%)	59 (85.5%)	56 (66.7%)	0.01
Esophageal varices bleeding	45 (29.4%)	23 (33.3%)	22 (26.2%)	0.33
Portal thrombosis	15 (9.8%)	10 (14.5%)	5 (6.0%)	0.07
Hepatocarcinoma	15 (9.8%)	7 (10.1%)	8 (9.5%)	0.89
Comorbidities (*N*, %)				
Diabetes mellitus	48 (31.4%)	26 (37.7%)	22 (26.2%)	0.13
Hypertension	71 (46.4%)	33 (47.8%)	38 (45.2%)	0.84
Heart failure	12 (7.8%)	5 (7.2%)	7 (8.3%)	0.82
Chronic kidney disease	5 (3.3%)	0 (0%)	5 (5.9%)	0.05
Immunosuppression (*N*, %)				
Corticosteroids	11 (7.2%)	2 (2.9%)	9 (10.7%)	0.08
Immunomodulators	3 (2.0%)	0	3 (3.6%)	0.11
Weight changes (*N*, %)				
Normal weight	113 (73.9%)	45 (65.2%)	68 (81.0%)	0.39
Underweight	4 (2.6%)	3 (4.3%)	1 (1.2%)	0.23
Overweight	11 (7.2%)	8 (11.6%)	3 (3.6%)	0.08
Obesity grade 1	12 (7.8%)	8 (11.6%)	4 (4.8%)	0.15
Obesity grade 2	6 (3.9%)	3 (4.3%)	3 (3.6%)	0.81
Obesity grade 3	7 (4.6%)	2 (2.9%)	5 (6.0%)	0.38
Active smoking (*N*, %)	23 (15.0%)	7 (10.1%)	16 (19.0%)	0.47
Alcohol consumption (*N*, %)				
Without consumption	74 (48.4%)	32 (46.4%)	42 (50.0%)	0.79
Moderate consumption	34 (22.2%)	17 (24.7%)	17 (20.2%)	0.60
Heavy consumption	31 (20.3%)	14 (20.3%)	17 (20.2%)	0.85
Laboratory testing at admission				
Hemoglobin (g/dL)	11.6 ± 2.5	11.6 ± 2.5	11.5 ± 2.6	0.94
Platelets (cell/mm^3^)	131.8 ± 186.0	103.7 ± 549.8	154.3 ± 245.8	0.09
Total bilirubin (mg/dL)	2.9 ± 3.4	3.1 ± 3.2	2.8 ± 3.6	0.53
INR	1.5 ± 0.4	1.5 ± 0.4	1.5 ± 0.5	0.76
Creatinine (mg/dL)	1.02 ± 1.01	0.9 ± 0.3	1.1 ± 1.3	0.10
Sodium (mEq/L)	136.9 ± 5.4	136.5 ± 5.8	137.3 ± 5.1	0.39
Albumin (g/dL)	3.2 ± 0.7	3.1 ± 0.7	3.3 ± 0.7	0.06
Ammonia (μmol/L)^a^	78.6 ± 54.1	111.2 ± 52.0	52.5 ± 41.5	0.02

a Ammonia levels were available for 18 patients.

ALD, alcohol liver disease; HH, hereditary hemochromatosis; INR, international normalized ratio; MASLD, metabolic dysfunction-associated steatotic liver disease; MELD-Na, Model of End-Stage Liver Disease sodium.

In the cohort, 69 (45%) patients used rifaximin. There were no significant differences between patients with use of rifaximin and those without in terms of age, gender, type of decompensation before admission, MELD-Na, and comorbidities. Likewise, there were no observed differences in the severity of cirrhosis (based on MELD-Na, *p* = 0.72) or in the etiologies of cirrhosis. However, the rifaximin group had more ascites (95.7% *versus* 85.7%, *p* = 0.04), overt HE (73.9% *versus* 40.5%, *p* < 0.001), and esophageal varices (85.5% *versus* 66.7%, *p* = 0.01) (Table 1). The cohort had a history of diabetes in 48 (31.4%), hypertension in 71 (46.4%), overweight 11 (7.2%), obesity 27 (16.3%), and heart failure in 12 (7.8%). Chronic kidney disease was observed in five (3.3%) cases, with a higher prevalence in the group that used rifaximin (0% *versus* 5.9%, *p* = 0.045). A total of 65 (42.5%) of patients had active alcohol intake, which was considered heavy in 31 (20.2%) of them, despite a prior diagnosis of cirrhosis. The use of corticosteroids and immunomodulators did not show significant differences (Table 1).

The mean follow-up was 32 months, 38.8 months for the rifaximin group, and 26.5 months for the control group. The main causes of cessation of follow-up in the rifaximin group were liver transplantation (29%) and loss of patient contact (29%).

### Impact of rifaximin use on infections

**Figure 2.**
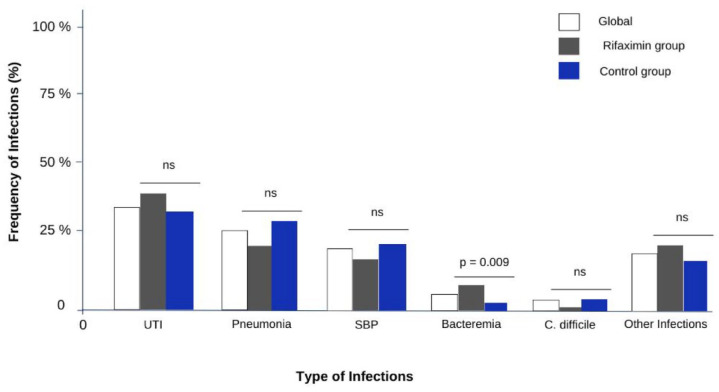
Frequency and type of infections (events). The diagram shows the percentage of patients with infections and the type of infections. No significant differences were observed between the groups in UTI (*p* = 0.1), pneumonia (*p* = 0.1), SBP (*p* = 0.6), *C. difficile* (*p* = 0.1), and other infections (*p* = 0.4). A significant difference was observed in Bacteremia (*p* = 0.009). *C. difficile, Clostridioides difficile*; ns, no statistically significant; SBP, spontaneous bacterial peritonitis; UTI, urinary tract infection.

A total of 85 patients with infectious events were recorded, of which 48 patients (56.4%) belonged to the control group, and 37 patients (43.6%) to the rifaximin group, and a total of 164 infectious events were registered, 87 (53%) from the rifaximin group and 77 (47%) from the control one. The main infectious events were represented by UTIs in 62 (37.8%), pneumonia in 38 (23.2%), SBP in 25 (15.2%), bacteremia in 8 (4.9%), and other infections in 28 (17.1%). Incidence of bacteremia was more frequent in the rifaximin group than in the control group (7.8% *versus* 2.3%, *p* = 0.09). No statistically significant differences were found between the groups in other infections (Figure 2). Only three subjects (3.4%) presented *C. difficile* diarrhea, all of them in the control group. In addition, in ‘other causes’, more than 50% in both groups were cases of cellulitis.

In the Poisson regression analysis within the non-adjusted model, the utilization of rifaximin showed no significant alteration in infection rates [IRR = 1.09, 95% confidence interval (CI) (0.8–1.4); *p* = 0.57]. However, upon adjusting the model for the defined confounding variables, we identified a substantial effect of rifaximin usage on infection rates [IRR = 0.64, 95% CI (0.47–0.89); *p* = 0.008] (Table 2). Later, the propensity score matching tool was used to balance the groups due to their initial differences. The analysis revealed an IRR of 0.55 [95% CI (0.4–0.77); *p* = 0.001]. An adjustment was also made for the use of corticosteroids and other prophylactic antibiotics, and this adjustment did not change the results.

**Table 2.** Impact of rifaximin use on infections, Poisson regression analysis.

Variables	Multivariate analysis		
	IRR	95% CI	*p* Value
Rifaximin use	0.64	0.472–0.891	0.008
Alcohol intake	0.96	0.836–1.112	0.621
Esophageal varices bleeding	1.04	0.715–1.530	0.814
Hepatic encephalopathy at admission			
Grade 1	1.73	1.080–2.785	0.023
Grade 2	1.80	1.159–2.810	0.009
Grade 3	5.44	2.061–14.354	0.001
Grade 4^a^	0	0	0
Child–Pugh at admission^b^			
B	1.16	0.768–1.755	0.478
C	1.48	0.883–2.498	0.135

a No EH Grade 4 was reported.

b Child Pugh Grade A was considered as the reference/baseline for the analysis in the model.

CI, confidence interval; IRR, internal rate of return.

### Impact of rifaximin on mortality

**Figure 3.**
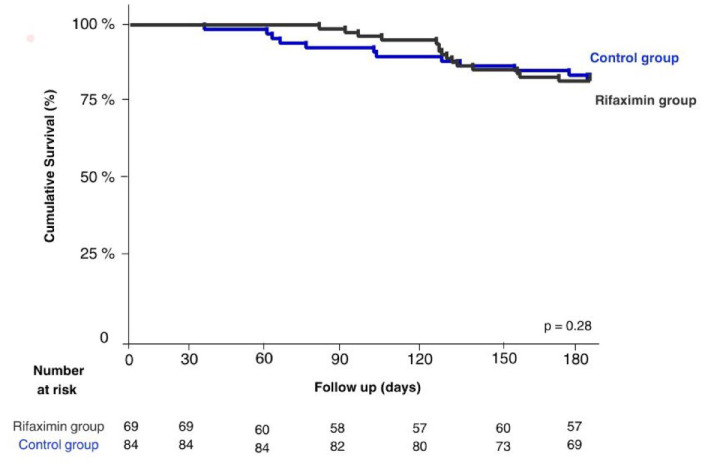
Survival of patients depending on the use of rifaximin. The graph illustrates a Kaplan–Meier curve depicting the survival of patients based on their use or non-use of rifaximin (*p* = 0.28).

**Figure 4.**
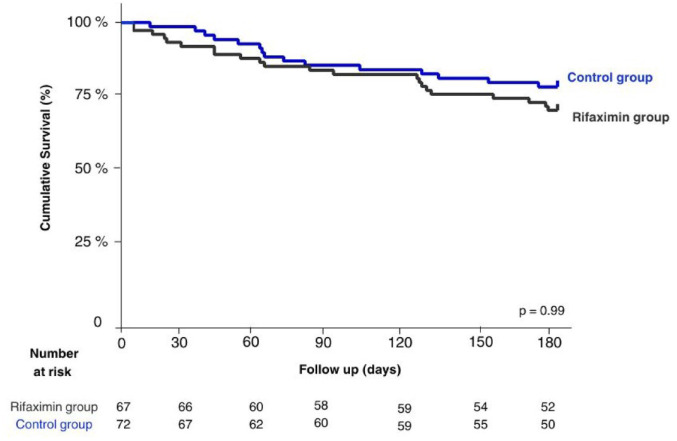
Infections-free survival of patients depending on the use of rifaximin. The graph illustrates a Kaplan–Meier curve depicting infections-free survival of patients based on their use or non-use of rifaximin (*p* = 0.99).

A total of 25 patients died during the follow-up. The main causes of mortality in both groups were multiple organ failure (20%), sepsis (12%), and renal failure (8%). No differences were observed in transplant-free survival [95% CI (0.4–2.1), *p* = 0.99] (Figure 3) nor infection-free survival [95% CI (0.7–2.7), *p* = 0.28] (Figure 4) through Kaplan–Meier analysis. In the mortality analysis conducted using a GLM, an impact was observed; however, it was not statistically significant in the unadjusted model [IRR = 2.16, 95% CI (1.0–4.0), *p* = 0.045] or in the adjusted model [IRR = 1.9, 95% CI (0.9–4.0), *p* = 0.090] (Table 3).

**Table 3.** Impact of rifaximin in mortality, GLM.

Variables	Multivariate analysis		
	IRR	95% CI	*p* Value
Rifaximin use	1.90	0.903–4.030	0.090
Infections	1.48	1.189–1.857	0.001
Hepatic encephalopathy at admission			
Grade 1	1.42	0.583–3.488	0.435
Grade 2	0.19	0.028–1.376	0.102
Grade 3	2.13	0.314–14.522	0.437
Grade 4^a^	–	–	–
Hepatic encephalopathy at end of follow-up^a^			
Grade 1	2.54	0.561–11.573	0.225
Grade 2	2.23	0.547–9.110	0.262
Grade 3	1.24	0.207–7.474	0.810
Child–Pugh at admission^b^			
B	0.70	0.300–1.638	0.413
C	0.52	0.184–1.507	0.232

a No EH Grade 4 was reported.

b Child Pugh Grade A was considered as the reference/baseline for the analysis in the model.

CI, confidence interval; GLM, generalized linear model; IRR, internal rate of return.

### Adherence to therapy and adverse effects

Regarding the use of rifaximin, throughout the study, 141 (82.6%) patients maintained rifaximin use during follow-up, and 12 (17.4%) patients discontinued it, of which 9 restarted it early, and only 3 patients kept the drug suspended. Among the reasons behind discontinuation, 2 were due to poor adherence, 5 to medical indication, and 4 to economic factors. During the study, no adverse effects were reported.

## Discussion

Infections substantially contribute to morbidity and mortality among cirrhotic patients. Therefore, strategies aimed at reducing their impact and frequency in this population are crucial.^1,5,7^ HE is a frequent complication of cirrhosis, for which rifaximin is commonly employed. This therapy is associated with minimal adverse effects and holds potential benefits not only in the management of this condition but also in addressing its broader implications.^14,22^ The utilization of rifaximin appears to offer benefits in mitigating SBP, gastrointestinal bleeding (including variceal bleeding), and HRS.^12,16,23^ Furthermore, it has demonstrated advantages in treating bacterial infections and, specifically, *C. difficile*-associated diarrhea.^12,17,24^ However, the current evidence is limited and sparse, which has precluded the establishment of comprehensive recommendations regarding its usage. In this registry-based cohort study comprising 153 patients with cirrhosis and HE, we observed that the utilization of rifaximin significantly reduces infections in these patients, with minimal adverse effects. Nevertheless, despite observing a trend, we were unable to demonstrate that this reduction in infections has a significant impact on mortality.

Bacterial infections contribute to a fourfold increase in the likelihood of death among patients with advanced or decompensated cirrhosis, resulting in a mortality rate of 30% at 1 month and 63% at 1 year following the infection.^25^ Common infection types observed in cirrhosis patients encompass SBP (25–31%), UTI (20–25%), pneumonia (15–21%), bacteremia (12%), and soft tissue infection (11%).^26^ In our study, infections were frequent, with a higher proportion of UTIs (37.8%) and SBP (15.2%), and bacteremia (4.9%) were less common compared to prior evidence. Meanwhile, the incidence of pneumonia and soft tissue infections (23.2% and 9.1%, respectively) closely resembled previous reports.

The use of rifaximin appears to play a significant role in reducing the frequency of infections in cirrhotic patients. An Italian observational study demonstrated a significantly lower risk of developing bacterial infections in patients treated with rifaximin [odds ratio (OR) 0.28; 95% CI (0.202–0.407); *p* < 0.001] compared to those not treated.^25^ Further evidence supports its efficacy in reducing the incidence of SBP. In a retrospective study involving 404 patients conducted in the United States, an impressive 72% reduction in the SBP rate was observed in the rifaximin group [hazard ratio 0.28; 95% CI (0.11–0.71); *p* = 0.007].^27^ In another systematic review and meta-analysis, which included 5 studies with 784 patients (186 receiving rifaximin, 598 without antibiotics), the OR for SBP was 0.34 [95% CI (0.11–0.99); *p* < 0.05] in patients treated with rifaximin.^28^ Some reports have also highlighted a positive effect of rifaximin in combination with norfloxacin compared to norfloxacin alone in the prevention of SBP. In a systematic review encompassing 6 studies with 973 patients, the pooled effect estimate indicated that the group receiving rifaximin plus norfloxacin had a lower incidence of SBP [relative risk (RR) 0.58; 95% CI (0.37–0.92); *p* = 0.02] and HE [RR 0.38; 95% CI (0.17–0.84); *p* = 0.02] than the group treated with norfloxacin alone.^26^ After conducting statistical adjustments, a significant reduction of up to 45% in infection incidence was observed in patients with cirrhosis and HE when using rifaximin. The use of rifaximin, a widely used medication in the management of HE, appears to play a vital role in reducing infections in cirrhotic patients with HE. In this context, it may be considered as a preferred option for HE management in patients at higher risk of infection, even in cases where formal indications for prophylactic antibiotics may not exist.

Several studies have successfully demonstrated the impact of rifaximin on mortality. In an Randomized Controlled Trial (RCT) involving 262 patients conducted in Egypt, which compared rifaximin to norfloxacin for secondary prevention of SBP, the mortality rate was significantly reduced in the rifaximin group (13.7% *versus* 24.4%, *p* = 0.044) compared to the norfloxacin group. Interestingly, no significant differences were observed in the causes of death between the two groups (*p* = 0.377).^29^ In the previously mentioned retrospective study involving 404 patients in the United States, the group treated with rifaximin also demonstrated a survival benefit without the need for a transplant compared to those not receiving rifaximin (72% *versus* 57%, *p* = 0.045).^27^ Additionally, in a multicenter, open, comparative pilot study focusing on patients with acute-on-chronic liver failure, the use of rifaximin in combination with corticosteroids *versus* corticosteroids alone showed a lower mortality rate in the rifaximin group, although it did not reach statistical significance (14.2% *versus* 30.9%, *p* = 0.15).^30^ In our study, we were unable to obtain significant mortality data, likely due to the small sample size and the exclusion of certain pathologies in the study design, such as hepatocellular carcinoma.

Interestingly, in addition to being a medication that reduces the infection rate and likely has some effect on the mortality of cirrhotic patients, rifaximin is also known for its remarkable safety profile. Numerous reports have described extremely low rates of adverse effects, with only a few reported cases of anaphylaxis and gastrointestinal symptoms.^14^ In our study, no adverse effects were reported. Another intriguing aspect is the high adherence rate to therapy observed in our study, with 82.6% of patients maintaining treatment throughout the follow-up period. This level of adherence is notably higher than what has been reported for the use of other antibiotics.^31^ It has been noted that one of the key factors influencing adherence is the frequency of adverse effects,^31^ which likely contributes to such high adherence rates with the use of rifaximin.

Our study has various limitations that deserve careful attention. To begin with, the retrospective design impedes our capacity to thoroughly manage potential confounding variables that could have impacted the results. Moreover, this design might result in an insufficient representation of the total infection count. Furthermore, the initial diagnosis of cirrhosis wasn’t validated through biopsy, and the determination of etiology and complications heavily leaned on clinical data and medical records. Also, dosification of rifaximin is slightly different compared to other countries, being at 400 mg TID compared to 550 mg BID in the United States. On the flip side, the limited sample size curtails the extent to which subgroup analysis and a comprehensive assessment of mortality can be applied effectively.

In conclusion, infections are common in patients with cirrhosis and have an impact on morbidity and mortality. Rifaximin is frequently used in patients with HE; however, benefits have been described in its use, reducing the risk of infections. Our results demonstrated a high frequency of infections in cirrhotic patients with HE, with UTI, pneumonia, and SBP being the main types. Interestingly, the use of rifaximin showed a significant reduction in infection rates in these patients. Future prospective studies are needed to confirm our findings and guide clinical management.

## Appendix

### Abbreviations

ALD Alcohol liver disease

CI Confidence interval

EVB Esophageal variceal bleeding

GLM Generalized linear model

HE Hepatic encephalopathy

HRS Hepatorenal syndrome

IRR Incidence rate ratio

MASLD Metabolic dysfunction-associated steatotic liver disease

MELD-Na Model of end-stage liver disease sodium

OR Odds ratio

RR Relative risk

SBP Spontaneous bacterial peritonitis

UTI Urinary tract infection

## Declarations

**Ethics approval and consent to participate:** The study protocol was approved by the Scientific Ethics Committee of Health Sciences of the Pontificia Universidad Católica de Chile, and a waiver of informed consent was obtained for the review of clinical records (ID: 210623028).

**Consent for publication:** Not applicable.

**Availability of data and materials:** The datasets generated and analyzed during the current study are not publicly available, but are available from the corresponding author upon reasonable request.

## References

[1] Tsochatzis EA Bosch J Burroughs AK. Liver cirrhosis. Lancet 2014; 383: 1749–1761. 24480518 10.1016/S0140-6736(14)60121-5

[2] Díaz LA Pages J Mainardi V , et al. Inpatient hepatology consultation: a practical approach for clinicians. Med Clin North Am 2023; 107: 555–565. 37001953 10.1016/j.mcna.2023.01.006

[3] Bruns T Zimmermann HW Stallmach A. Risk factors and outcome of bacterial infections in cirrhosis. World J Gastroenterol 2014; 20: 2542–2554. 24627590 10.3748/wjg.v20.i10.2542 PMC3949263

[4] Piano S Singh V Caraceni P , et al. Epidemiology and effects of bacterial infections in patients with cirrhosis worldwide. Gastroenterology 2019; 156: 1368–1380.e10. 10.1053/j.gastro.2018.12.005 30552895

[5] Volk ML Tocco RS Bazick J , et al. Hospital readmissions among patients with decompensated cirrhosis. Am J Gastroenterol 2012; 107: 247–252. 21931378 10.1038/ajg.2011.314 PMC3470789

[6] Piano S Tonon M Angeli P. Changes in the epidemiology and management of bacterial infections in cirrhosis. Clin Mol Hepatol 2021; 27: 437–445. 33504138 10.3350/cmh.2020.0329 PMC8273641

[7] Bajaj JS Kamath PS Reddy KR. The evolving challenge of infections in cirrhosis. N Engl J Med 2021; 384: 2317–2330. 34133861 10.1056/NEJMra2021808

[8] Hassner A Kletter Y Shlag D , et al. Impaired monocyte function in liver cirrhosis. Br Med J 1981; 282: 1262–1263. 6784806 10.1136/bmj.282.6272.1262 PMC1505383

[9] Patel VC Williams R. Antimicrobial resistance in chronic liver disease. Hepatol Int 2020; 14: 24–34. 31797303 10.1007/s12072-019-10004-1 PMC6994429

[10] Fernández J Prado V Trebicka J , et al. Multidrug-resistant bacterial infections in patients with decompensated cirrhosis and with acute-on-chronic liver failure in Europe. J Hepatol 2019; 70: 398–411. 30391380 10.1016/j.jhep.2018.10.027

[11] Kremer WM Gairing SJ Kaps L , et al. Characteristics of bacterial infections and prevalence of multidrug-resistant bacteria in hospitalized patients with liver cirrhosis in Germany. Ann Hepatol 2022; 27: 100719. 35460883 10.1016/j.aohep.2022.100719

[12] Caraceni P Vargas V Solà E , et al. The use of rifaximin in patients with cirrhosis. Hepatology 2021; 74: 1660–1673. 33421158 10.1002/hep.31708 PMC8518409

[13] Sanchez-Delgado J Miquel M. [Role of rifaximin in the treatment of hepatic encephalopathy]. Gastroenterol Hepatol 2016; 39: 282–292. 26545947 10.1016/j.gastrohep.2015.08.003

[14] Bass NM Mullen KD Sanyal A , et al. Rifaximin treatment in hepatic encephalopathy. N Engl J Med 2010; 362: 1071–1081. 20335583 10.1056/NEJMoa0907893

[15] Fu J Gao Y Shi L. Combination therapy with rifaximin and lactulose in hepatic encephalopathy: a systematic review and meta-analysis. PLoS One 2022; 17: e0267647. 10.1371/journal.pone.0267647 PMC9041837 35471992

[16] Salehi S Tranah TH Lim S , et al. Rifaximin reduces the incidence of spontaneous bacterial peritonitis, variceal bleeding and all-cause admissions in patients on the liver transplant waiting list. Aliment Pharmacol Ther 2019; 50: 435–441. 31169941 10.1111/apt.15326 PMC6816014

[17] Feuerstadt P Hong SJ Brandt LJ. Chronic rifaximin use in cirrhotic patients is associated with decreased rate of *C. difficile* infection. Dig Dis Sci 2020; 65: 632–638. 31440997 10.1007/s10620-019-05804-2

[18] Lv XY Ding HG Zheng JF , et al. Rifaximin improves survival in cirrhotic patients with refractory ascites: a real-world study. World J Gastroenterol 2020; 26: 199–218. 31988585 10.3748/wjg.v26.i2.199 PMC6962437

[19] Zeng X Sheng X Wang PQ , et al. Low-dose rifaximin prevents complications and improves survival in patients with decompensated liver cirrhosis. Hepatol Int 2021; 15: 155–165. 33385299 10.1007/s12072-020-10117-y

[20] Stoll AM Guido M Pence A , et al. Lack of access to rifaximin upon hospital discharge is frequent and results in increased hospitalizations for hepatic encephalopathy. Ann Pharmacother 2023; 57: 133–140. 35658580 10.1177/10600280221100537

[21] National Institute on Alcohol Abuse and Alcoholism (NIAAA). Drinking levels defined [Internet], https://www.niaaa.nih.gov/alcohol-health/overview-alcohol-consumption/moderate-binge-drinking (2023, accessed 6 August 2021).

[22] Vilstrup H Amodio P Bajaj J , et al. Hepatic encephalopathy in chronic liver disease: 2014 Practice Guideline by the American Association for the Study of Liver Diseases and the European Association for the Study of the Liver. Hepatology 2014; 60: 715–735. 25042402 10.1002/hep.27210

[23] Bajaj JS O’Leary JG Reddy KR , et al. Second infections independently increase mortality in hospitalized patients with cirrhosis: the North American consortium for the study of end-stage liver disease (NACSELD) experience. Hepatology 2012; 56: 2328–2335. 22806618 10.1002/hep.25947 PMC3492528

[24] Swartz MN. Clinical practice. Cellulitis. N Engl J Med 2004; 350: 904–912. 14985488 10.1056/NEJMcp031807

[25] Mariani M Zuccaro V Patruno SFA , et al. The impact of rifaximin in the prevention of bacterial infections in cirrhosis. Eur Rev Med Pharmacol Sci 2017; 21: 1151–1158. 28338174

[26] Menshawy A Mattar O Barssoum K , et al. Safety and efficacy of rifaximin in prophylaxis of spontaneous bacterial peritonitis: a systematic review and meta-analysis. Curr Drug Targets 2019; 20: 380–387. 30246636 10.2174/1389450119666180924145156

[27] Hanouneh MA Hanouneh IA Hashash JG , et al. The role of rifaximin in the primary prophylaxis of spontaneous bacterial peritonitis in patients with liver cirrhosis. J Clin Gastroenterol 2012; 46: 709–715. 22878533 10.1097/MCG.0b013e3182506dbb

[28] Goel A Rahim U Nguyen LH , et al. Systematic review with meta-analysis: rifaximin for the prophylaxis of spontaneous bacterial peritonitis. Aliment Pharmacol Ther 2017; 46: 1029–1036. 28994123 10.1111/apt.14361

[29] Elfert A Abo Ali L Soliman S , et al. Randomized-controlled trial of rifaximin *versus* norfloxacin for secondary prophylaxis of spontaneous bacterial peritonitis. Eur J Gastroenterol Hepatol 2016; 28: 1450–1454. 27512927 10.1097/MEG.0000000000000724

[30] Jiménez C Ventura-Cots M Sala M , et al. Effect of rifaximin on infections, acute-on-chronic liver failure and mortality in alcoholic hepatitis: a pilot study (RIFA-AH). Liver Int 2022; 42: 1109–1120. 35220659 10.1111/liv.15207 PMC9311407

[31] Endashaw Hareru H Sisay D Kassaw C , et al. Antibiotics non-adherence and its associated factors among households in southern Ethiopia. SAGE Open Med 2022; 10: 20503121221090472. 35465633 10.1177/20503121221090472 PMC9021478

